# Molecular Mechanism of Aflatoxin B_1_ Synthesis Related AfVerB Regulating the Development, AFB_1_ Biosyntheis and Virulence of *Aspergillus flavus* Mainly Through Its CYP Domain

**DOI:** 10.3390/jof11040293

**Published:** 2025-04-09

**Authors:** Kangfu Ye, Song Zhou, Dandan Wu, Dongmei Ma, Yanfang Yao, Chi Yang, Minghui Sun, Sile Yang, Wangzhuo Fu, Wenwen Xin, Jun Yuan, Zhenhong Zhuang, Yanling Yang

**Affiliations:** 1 Key Laboratory of Pathogenic Fungi and Mycotoxins of Fujian Province, Key Laboratory of Biopesticide and Chemical Biology of Education Ministry, Proteomic Research Center, School of Life Sciences, Fujian Agriculture and Forestry University, Fuzhou 350002, China; yekf828@163.com (K.Y.); zhousongfafu@163.com (S.Z.); kikimra@163.com (D.W.); 5220543002@fafu.edu.cn (Y.Y.); yc113078@163.com (C.Y.); xwyy959526@gmail.com (S.Y.); Fuwangzhuo@foxmail.com (W.F.); 2 College of Animal Sciences, Fujian Agriculture and Forestry University, Fuzhou 350002, China; 6220623006@fafu.edu.cn (D.M.); hnmhsun@126.com (M.S.); 3 State Key Laboratory of Pathogen and Biosecurity, Institute of Microbiology and Epidemiology, Academy of Military Medical Sciences (AMMS), Beijing 100071, China; xinww@hotmail.com

**Keywords:** *Aspergillus flavus*, AFB_1_, AfVerB, CYP domain, fungal drug resistance

## Abstract

*Aspergillus flavus* and its secondary metabolites aflatoxins pose a significant threat to the health of humans, animals, and plants. Therefore, there is an urgent need to control *A. flavus* contamination. AfverB plays a key role in the aflatoxin gene cluster; however, its function and mechanism in fungal development and virulence remain poorly understood. In this study, we constructed *afVerB* gene deletion mutants (∆*afVerB*−1 and ∆*afVerB*−2) and two CYP domain mutants (*afVerB*^∆D1^ and *afVerB*^∆D2^) through homologous recombination. Phenotype analysis revealed that, via its two CYP domains, AfVerB is deeply involved in fungal morphogenesis and aflatoxin synthesis. Insect and crop colonization models revealed that AfVerB plays a key role in the fungus’s ability to infect hosts, and stress experiments discovered that AfVerB plays a significant role in the response to various environmental stresses, which explains why AfVerB is a key factor in fungal infection to some extent. RT-qPCR analysis demonstrated that AfVerB performs its bio-function through corresponding regulatory factors. We ultimately discovered that AfVerB is deeply involved in cell membrane stress stability, thereby participating in the regulation of fungal drug resistance (sensitive to AMB and resistant to VOR in this study). The CYP domain of AfVerB, particularly its second CYP domain, is crucial for the execution of its biological functions. This study elucidated the regulatory mechanisms by which AfVerB regulates fungal pathogenicity and aflatoxin biosynthesis, providing potential strategies for controlling *A. flavus* and its aflatoxin contamination.

## 1. Introduction

*Aspergillus* spp. Are widely distributed all over the world, and a number of species produce mycotoxins, acting as pathogens that seriously jeopardize the health of plants and animals [1]. In addition, after *Aspergillus fumigatus*, *A. flavus* is the second most common cause of human aspergillosis [2]. Aflatoxin B_1_ (AFB_1_) is the most toxic and prevalent aflatoxin, and it has been classified as a group 1 carcinogen by the International Agency for Research on Cancer [3]. In recent years, research on aflatoxin synthesis has received much attention in attempts to reduce their impact on agricultural economies and human health [4]. Despite a series of advances in this field, our understanding of the molecular regulatory mechanisms of aflatoxin biosynthesis by *A. flavus* is still limited. Therefore, there is an urgent need to reveal the regulatory mechanisms on aflatoxin biosynthesis and fungal virulence in *A. flavus*.

Aflatoxins are toxic and carcinogenic secondary metabolites derived from polyketide compounds, and their synthesis mainly involves at least 27 enzymes, which constitute a 70 kb gene cluster in the fungal genome, modulated by two transcriptionally regulators, AflR and AflS [5,6,7]. In *A. flavus*, AflR regulates fungal development, nucleus formation, and toxin synthesis [8,9]. AflR and AflS form a complex, in which AflS does not exhibit DNA-binding ability but reduces the DNA-binding affinity of AflR [10]. The deletion of the *aflN* gene from the gene cluster results in blocked toxin synthesis and is associated with fungal responses to environmental oxidative stress and reactive oxygen species in cells [11]. In the cluster, *aflX* encodes an enzyme involved in the conversion of Versicolorin A to Demethylsterigmatocystin, *aflO* regulates aflatoxin synthesis and fungal pathogenicity through lysine acetylation, and *aflQ* regulates aflatoxin production through its m^6^A modification [5,12,13]. It has been shown that aflatoxin synthesis is also dependent on cytochrome p450 monooxygenase [14]. AfVerB is involved in the transformation of norsolorinic acid to Averufin (AVN), which is crucial for the synthesis of aflatoxin. It is also involved in methylation modification and mRNA stability modulation, and RNA-Seq and RT-qPCR have revealed that AfVerB takes part in aflatoxin synthesis regulated by the methyltransferase AflIme4 [15,16,17]. However, the biological function of AfVerB in fungal virulence and aflatoxin biosynthesis remains unclear.

Currently, the primary antifungal agents employed in the treatment of invasive aspergillosis are polyenes, azoles, and echinocandins. Among these, voriconazole and amphotericin B play crucial roles in the prevention and clinical management of invasive aspergillosis [18,19]. Previous research has indicated that azoles and other fungicides can effectively suppress the incidence of diseases and the production of mycotoxins caused by fungi from the genera *Aspergillus* [20,21]. In the context of *A. flavus*, a robust correlation has recently been unveiled between azole resistance and genes associated with the aflatoxin biosynthesis gene cluster [22]. Nevertheless, the biological functions of AfVerB in modulating the drug resistance of *A. flavus* remain to be elucidated.

In this study, we investigated the biological functions of AfVerB in fungal development, toxin biosynthesis, pathogenicity, and responses to diverse environmental stresses and drug resistance by constructing AfVerB mutants and key domain mutants through crop and insect models. This work provides theoretical support for elucidating the significance of this gene in the control of *A. flavus* contamination.

## 2. Materials and Methods

### 2.1. The A. flavus Strains and Media Used in This Study

The *A. flavus* strains used in this study are listed in Table S1. The media used for the incubation of fungal strains in this study are listed in Table S2.

### 2.2. The Construction of A. flavus Strains

All fungal mutants in this article were constructed through homologous recombination [23]. *afVerB* knockout strain (∆*afVerB*) was constructed as shown in Figure S1A. Three primer pairs (*afVerB*-AF and *afVerB*-AR, *afVerB*-BF and *afVerB*-BR, and *pyrG*-F and *pyrG*-R) were used in the amplification of the upstream region (1901 bp), the downstream region (1811 bp), and the selective marker *pyrG*, respectively. The fusion of the above three fragments was performed with primers *afVerB* -NF and *afVerB* -NR. Finally, the ∆*afVerB* strain was obtained by the introduction of the fused fragment into CA14 PTS by polyethylene glycol-mediated transformation. Three pairs of primers (*afVerB*-AF and P801, P1020 and *afVerB*-BR, and *afVerB*-OF and *afVerB*-OR) were used to verify the ∆*afVerB* strain by amplifying the upstream homology arm fragment (AP), the downstream homology arm (BP), and open reading frames (ORFs). Transformants with AP and BP but without ORF fragments were confirmed as ∆*afVerB* strains. The domains of AfVerB were predicted by NCBI’s CCD database (https://www.ncbi.nlm.nih.gov/Structure/cdd/cdd.shtml, accessed on 28 August 2024), and the construction of domain deletion strains followed the protocol used for ∆*afVerB* preparation, as shown in Figure S1B,C.

### 2.3. Phenotype Analysis

The phenotype of the mutants was observed according to the previous study [24]. WT, ∆*afVerB*−1, ∆*afVerB*−2, *afVerB*^∆D1^, and *afVerB*^∆D2^ strains were incubated in the dark on PDA, YES, and GMM media at 37 °C. After 4 d, the diameter of each colony was measured. Conidia from each sample were collected and washed with a spore elution solution (0.05% Tween-20 and 7% DMSO). The collected conidia were analyzed and counted with a hemocytometer under a light microscope (Leica, Heerbrugg, Germany). For sclerotia analysis, CM medium was prepared to induce mycorrhizal formation, and 10^4^ spore suspensions were inoculated and incubated in the dark at 37 °C. After 7 d of incubation, conidia and hyphae were washed away by spraying with 75% ethanol, and the sclerotia were counted under a light microscope (Leica, Heidelberg, Germany). The experiment was performed in three replicates.

### 2.4. Analysis of AFB_1_ Production

Aflatoxin yield was monitored according to the previous protocol with minor modifications [25]. Spores (10 μL, 10^7^ conidia/mL) of WT, ∆*afVerB*−1, ∆*afVerB*−2, *afVerB*^∆D1^, and *afVerB*^∆D2^ fungal strains were inoculated in 10 mL of PDB and incubated in the dark at 29 °C for 7 d. The bottom 4 mL of the medium was mixed with an equal amount of methylene chloride, and the mixtures were shaken at 29 °C for 1 h at 150 rpm. Then, the lower 3 mL of liquid was transferred into a 5 mL centrifuge tube and dried in a fume hood. Then, aflatoxins were re-dissolved with 100 μL of methylene chloride, and 10 μL of the solution was analyzed by thin-layer chromatography (TLC) (Haiyang Chemical, Qingdao, China). The results were recorded using a UV gel imaging system (Beijing Oriental Science and Technology Development Ltd., Beijing, China). The experiment was performed in three replicates.

### 2.5. Crop Colonization Analysis

The research was conducted based on the previous methods [26]. Six peanut and corn kernels of uniform size and good growth were sterilized by soaking in 0.05% sodium hypochlorite for 3 min, followed by a thorough rinsing with alcohol and sterile water, and then inoculated with the spore suspensions (10^7^ conidia/mL) of WT, ∆*afVerB*−1, ∆*afVerB*−2, *afVerB*^∆D1^, and *afVerB*^∆D2^ strains. The inoculated kernels were incubated at 29 °C for 6 d. The infested host kernels were then collected in a 50 mL centrifuge tube, vortexed in 10 mL of ddH_2_O, and 1 mL of spore suspension was aspirated to count the number of spores with a hemocytometer. Then, 10 mL of dichloromethane was added to the centrifuge tube to extract the aflatoxins, and the extracted aflatoxins were analyzed by thin-layer chromatography. The experiment was peformed in three replicates.

### 2.6. Stress Analysis

The fungal susceptibility reaction to various stresses was analyzed based on the previous methods [27]. To evaluate the role of AfVerB in the fungal resistance against environmental stresses, fungal spores (10^7^) were inoculated on PDA under a variety of inhibitors (including the cell membrane inhibitor SDS, osmotic stress NaCl, cell wall inhibitor CR, the DNA damaging agent MMS, and oxidative stress MSB). The diameters of fungal colonies were determined at the 3rd d after inoculation. Finally, the inhibition rate was calculated according to the following formula: inhibition rate = (diameter of colony without inhibitor—diameter of colony with inhibitor)/diameter of colony without inhibitor. The experiment was performed in three replicates.

### 2.7. Insect Infection Model

The role of AfVerB in the fungal infection of wax borers (*Galleria mellonella*) was assayed according to the previously described method [28]. Ten *G.mellonella* were selected for each experimental group. Each *G mellonella* was injected with 5 μL of spore suspension (10^7^ conidia/mL). *G.mellonella* larvae injected with saline served as the control group. The injected wax borers were incubated in a dark at room temperature, and the survival rate was recorded for the next 5 d. The dead larvae were collected and placed at 29 °C for 7 d, after which conidiation ability and AFB_1_ yield were analyzed according to the aforementioned protocols. The experiment was performed in three replicates.

### 2.8. Agar Spotting Assays

The role of AfVerB in fungal drug resistance was analyzed according to a previous protocol [29]. To test the role of AfVerB in fungal drug resistance, the sensitivity of WT, Δ*afVerB*−1 and Δ*afVerB*−2 strains to AMB and VOR was analyzed, Müller-Hinton medium was supplemented with 0.67 μg/mL AMB and 0.25 μg/mL VOR. Then, 1 μL portions of conidial suspensions (2 × 10^6^, 2 × 10^5^, 2 × 10^4^, 2 × 10^3^ conidia/mL) of the indicated fungal strains were spotted onto the plates with corresponding media, then grown at 37 °C for 48 h, and the results were observed and documented. The experiment was repeated three times.

### 2.9. RT-qPCR Assays

Fungal spores (10^6^/mL) were cultured in PDB for 48 h. Mycelium was ground into powder with liquid nitrogen, and each 0.1 g of mycelium powder was lysed in 1 mL of TRIzol Total RNA Extraction Reagent (TakaRa Bio, Inc., Kusatsu, Japan) for 30 min. Total RNA was then extracted according to a previous protocol with a few modifications [25]. The first-strand cDNA was synthesized using the HiScript III enzyme mixture (Vazyme Biotechnology, Nanjing, China) according to the manufacturer’s instructions. RT-qPCR analyses were performed with the Quantstudio1+PCR system (Applied Biosystems, Inc., Walthman, MA, USA). The primer sequences used for RT-qPCR in this study are shown in Table S3. The expression level of *β*-*tubulin* was used as the internal reference. The relative expression levels of the target genes were calculated following the formula 2^−∆∆Ct^. All experiments were repeated three times.

### 2.10. Statistical Analysis

All data in this study are expressed as mean ± standard deviation. Statistical analysis was performed using GraphPad Prism 8.0.2 and the one-way ANOVA with Tukey’s test. A difference was considered to be statistically significant when *p* < 0.05. Error bars indicate standard error from at least three repetitions.

## 3. Results

### 3.1. Bioinformatics Analysis and Strain Construction

**Figure 1:**
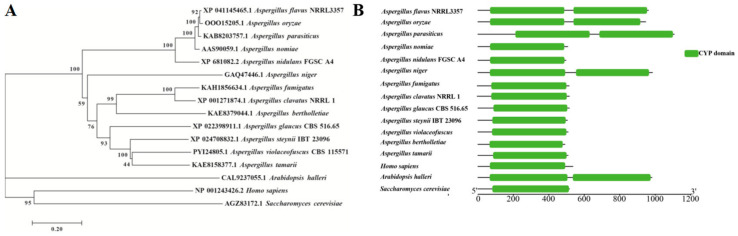
Bioinformatics analysis of AfVerB. (**A**) Evolutionary analysis of AfVerB. Homologous proteins of AfVerB from 16 model species, including *A. flavus*, *A. bertholletiae*, *A. clavatus*, *A. glaucus*, *A. steynii*, *A. tamarii*, *A. violaceofuscus*, *A. nidulans*, *A. niger*, *A. nomiae*, *A. parasiticus*, *A. oryzae*, *A. fumigatus*, *S. cerevisiae*, *H. sapiens*, and *A. halleri*, were aligned and the evolutionary tree was constructed using MEGA 7.0. (**B**) Domain analysis of AfVerB and its homologous proteins. Green bars represents the CYP domain.

To reveal the potential biological functions of AfVerB in *A. flavus*, the gene encoding *afVerB* (XP 041145465.1) was downloaded from NCBI (http://www.ncbi.nlm.nih.gov, accessed on 28 August 2024). The orthologs of *afVerB* were identified by BLAST in NCBI from 15 other species (*A. bertholletiae*, *A. clavatus*, *A. glaucus*, *A. steynii*, *A. tamarii*, *A. violaceofuscus*, *A. nidulans*, *A. niger*, *A. nomiae*, *A. parasiticus*, *A. oryzae*, *A. fumigatus*, *S. cerevisiae*, *H. sapiens*, and *A. halleri*). The evolutionary relationships of these homologous genes were constructed by MEGA 7.0 (Figure 1A), which showed that AfVerB is more closely related to *A. oryzae* VerB, and VerB is conserved among *Aspergillus* spp. The domains of VerB were predicted through CCD (https://www.ncbi.nlm.nih.gov/Structure/cdd/cdd.shtml, accessed on 28 August 2024) and visualized by tbtools (Toolbox for Biologists v2.149), which found that they all contain the conserved cyp domains. All the species of *Aspergillus* listed in the evolutionary tree bear a CYP domain. Among them, the VerB proteins of *A. flavus*, *A*. *oryzae*, *A. niger*, and *A. parasiticus* contain two CYP domains (Figure 1B). The above results suggest that AfVerB is conserved and may play important roles in the development and virulence of *A. flavus*.

The *afVerB* deletion mutant, including ∆*afVerB*−1, ∆*afVerB*−2, were constructed according to the protocol shown in Figure S1A. The transformants were verified through diagnostic PCR, and the results showed that both AP and BP fragments could be amplified from ∆*afVerB*−1 and ∆*afVerB*−2, but no ORF fragment was detected in both mutants (Figure S2A,B), which reflected that *afVerB* had been successfully deleted from both mutants. Further, the expression level of *afVerB* was further monitored by RT-qPCR, and the results showed that *afVerB* could not be detected from both ∆*afVerB*-1 and ∆*afVerB*-2 mutants, which meant that both *afVerB* deletion mutants were constructed (Figure S2E). The domain deletion strains (a*fVerB*^∆D1^ and *afVerB*^∆D2^) were constructed and tested following the above method (Figure S2C,D), and were further confirmed through sequencing by Fuzhou Sunya Biotechnology Co., Ltd. (Fuzhou, China) (Figure S2F,G).

### 3.2. AfVerB Is Deeply Involved in Fungal Growth and Conidiation Through Its Both CYP Domains

**Figure 2:**
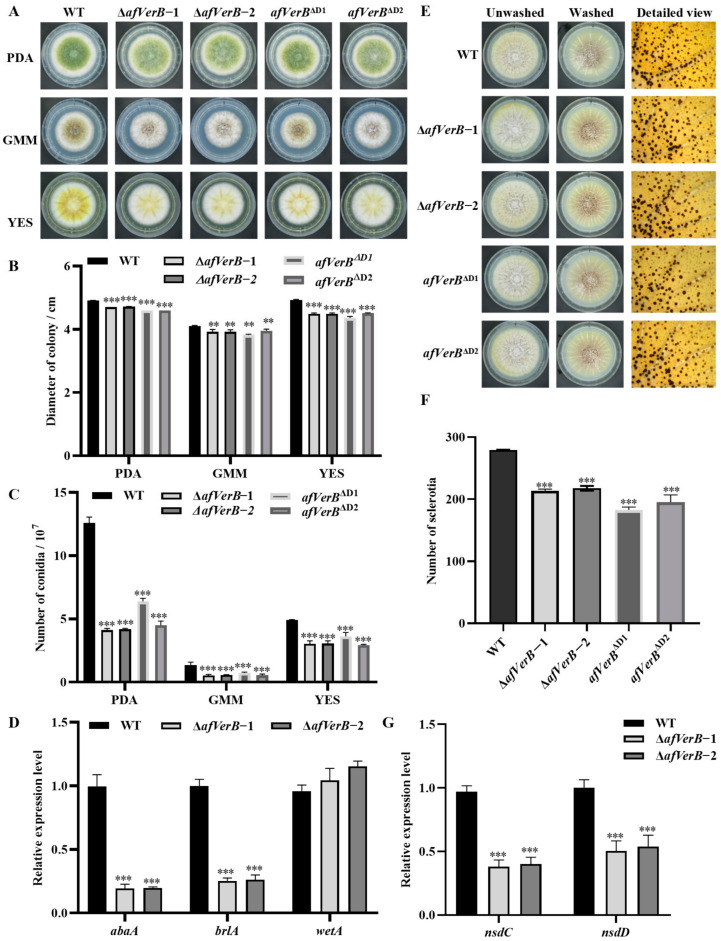
AfVerB plays a crucial role in the development of *A. flavus.* (**A**) The growth of WT, ∆*afVerB*−1, ∆*a*f*VerB*−2, ∆*a*f*VerB*^D1^, and ∆*a*f*VerB*^D2^ strains on PDA, GMM, and YES media in the dark at 37 °C for 4 d. (**B**) Statistical analysis of the colony diameters of the aforementioned strains. (**C**) Statistical analysis of the spore number of the aforementioned strains. (**D**) The relative expression levels of *abaA*, *brlA*, and *wetA* in WT, ∆*a*f*VerB*−1, and ∆*a*f*VerB*−2 strains. (**E**) Sclerotia formation in the aforementioned fungal strains. Hyphae and conidia were washed off with 75% ethanol, and the detailed images before and after washing are provided. (**F**) Statistical analysis of the sclerotia produced by the aforementioned strains. (**G**) Statistical analysis of the relative expression levels of *nsdC* and *nsdD* in WT, ∆*a*f*VerB*−1, and ∆*a*f*VerB*−2 strains. **, *** means significant differences at *p* < 0.01 and *p* < 0.001, respectively.

In this study, in order to investigate the effect of VerB on the growth of *A. flavus* as well as conidia production, the spore suspension of WT, ∆*afVerB*−1, ∆*afVerB*−2, *afVerB*^∆D1^, and *afVerB*^∆D2^ strains were diluted to the same concentration and then cultured on PDA, YES, and GMM media for 4 d in dark at 37 °C. Photographs were taken and the diameter of each colony was measured. The number of spores was counted by a hemocytometer. The results showed that the colonial diameters of ∆*afVerB*−1, ∆*afVerB*−2, *afVerB*^∆D1^, and *afVerB*^∆D2^ were significantly smaller compared to that of the WT strain (Figure 2A,B), and the conidia number of ∆*afVerB*−1, ∆*afVerB*−2, *afVerB*^∆D1^, and *afVerB*^∆D2^ was significantly lower (Figure 2A,C). Further RT-qPCR showed that the deletion of *AfVerB* reduced conidia production by downregulating the expression levels of conidiation-related transcriptional factors *abaA* and *brlA* (Figure 2D). The above results suggest that *AfVerB* plays a crucial role in *A. flavus* conidiation.

### 3.3. AfVerB Is Involved in the Formation of Sclerotia via Both CYP Domains

Sclerotia is a kind of resistance structure formed by *A. flavus* to resist harsh external environments. In order to investigate the effect of *afVerB* on sclerotia formation, the spore suspension of WT, ∆*afVerB*−1, ∆*afVerB*−2, *afVerB*^∆D1^, and *afVerB*^∆D2^ strains were diluted to the same concentration and then incubated on CM medium at 37 °C in the dark for 7 d. The results showed that the sclerotia number of ∆*afVerB*−1, ∆*afVerB*−2, *afVerB*^∆D1^, and *afVerB*^∆D2^ was significantly reduced compared to the WT strain (Figure 2E,F). In order to reveal the potential pathway through which AfVerB regulates the formation of sclerotia, we examined the expression levels of transcriptional factors *nsdC* and *nsdD* genes by RT-qPCR. The results indicated that the relative expression levels of *nsdC* and *nsdD* were significantly reduced in both ∆*afVerB*−1 and ∆*afVerB*−2 compared with the WT strain (Figure 2G). The above results reveals that AfVerB plays an important role in sclerotia formation.

### 3.4. AfVerB Affects AFB_1_ Biosynthesis Through Its Second CYP Domain

**Figure 3:**
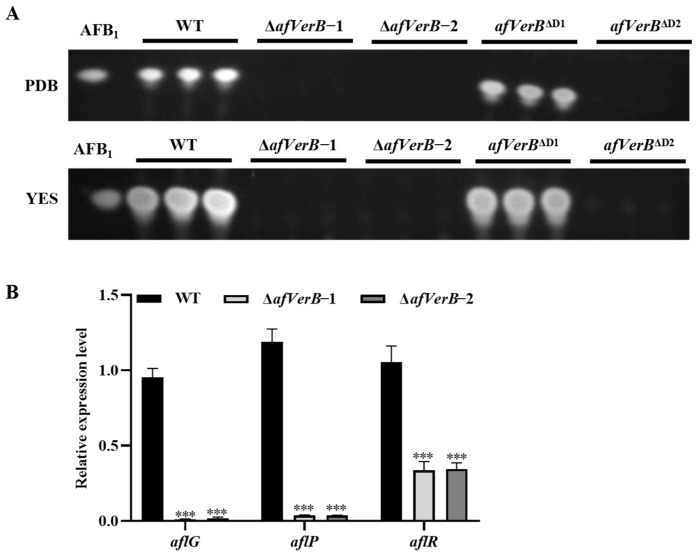
AfVerB plays a key role in AFB_1_ biosynthesis. (**A**) The biosynthesis of AFB_1_ in ∆*afVerB*−1, ∆*afVerB*−2, *afVerB*^∆D1^, and *afVerB*^∆D2^ strains were detected by TLC after being grown PDB and YES at 29 °C in the dark for 7 d. (**B**) Statistical analysis of the relative expression levels of *aflG*, *aflP*, and *aflR* in WT, ∆*afVerB*−1, and ∆*a*f*VerB*−2 strains. *** indicates *p* < 0.001.

In order to investigate the effect of *afVerB* on AFB1 biosynthesis, the spores of WT, ∆*afVerB*−1, ∆*afVerB*−2, *afVerB*^∆D1,^ and *afVerB*^∆D2^ strains were diluted to the same concentration and incubated on PDB and YES for 7 d in the dark at 29 °C. The production of AFB_1_ from the above fungal strains was analyzed by TLC. It was found that AFB_1_ could not be detected in ∆*afVerB*−1, ∆*afVerB*−2, and *afVerB*^∆D2^, suggesting that *afVerB* is indispensable for aflatoxin biosynthesis, and that its second CYP domain is the key domain in this regulation process (Figure 3A). Further RT-qPCR analysis of the expression levels of genes in the aflatoxin gene cluster showed that *aflR*, *aflG,* and *aflP* were all significantly down-regulated in both ∆*afVerB*−1 and ∆*afVerB*−2 strains compared to the WT strain (Figure 3B). These results suggested that, through its second CYP domain, AfVerB plays a key role in AFB_1_ biosynthesis by regulating the aflatoxin gene cluster.

### 3.5. AfVerB Is Deeply Involved in the Colonization of Crop Grains by A. flavus

**Figure 4:**
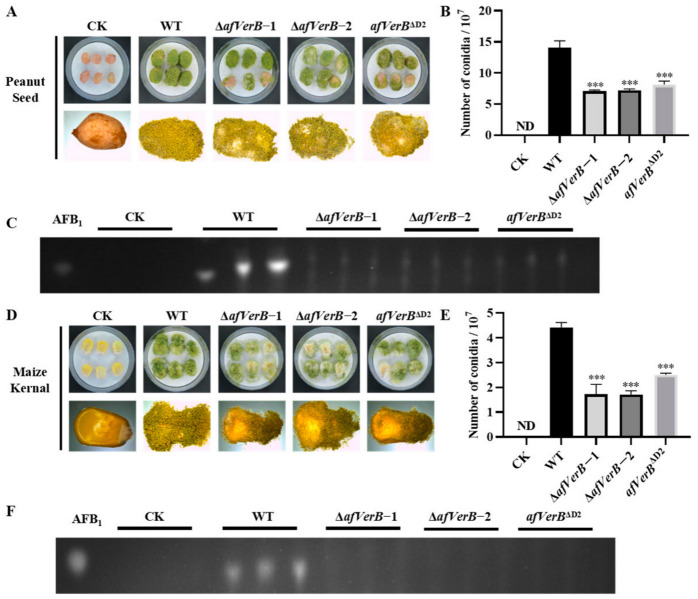
The role of AfVerB in the ability of *A. flavus* to colonize host crops. (**A**) The colonization of WT, ∆*afVerB*−1, ∆*afVerB*−2, and *afVerB*^∆D2^ strains on peanut seeds at 29 °C in dark for 5 d. (**B**) Statistical analysis of the spore numbers from the aforementioned fungal strains on peanut seeds. (**C**) The TLC analysis of the AFB_1_ production from the aforementioned fungal strains on peanut seeds. (**D**) The colonization of WT, ∆*afVerB*−1, ∆*afVerB*−2, and *afVerB*^∆D2^ strains on corn kernels. (**E**) Statistical analysis of the spore numbers from the aforementioned fungal strains on corn kernel. (**F**) The TLC analysis of the AFB_1_ production from the aforementioned fungal strains on corn kernels. *** indicates *p* < 0.001.

In order to elucidate the role of the *afVerB* in the colonization of *A. flavus* on the kernel of peanut and corn, grains of both corn and peanut were inoculated with the spore suspension of the above fungal strains and subsequently incubated in dark at 29°C for 5 d.The spores produced on peanut and corn kernels were then calculated, and the results showed that significantly fewer spores were produced on peanut kernels inoculated with ∆*afVerB*−1, ∆*afVerB*−2, and *afVerB*^∆D2^ than in the WT strain group (Figure 4A,B). Further TLC analyses showed that little AFB_1_ was detected from corn and peanut kernels in the ∆*afVerB*−1, ∆*afVerB*−2, and *afVerB*^∆D2^ groups, which was dramatically less than that of the WT group (Figure 4C). Similar results were observed in the corn kernels (Figure 4D–F). The above results suggest that the absence of AfVerB or its second CYP domain significantly reduces the ability of *A. flavus* to colonize crop kernels.

### 3.6. AfVerB Is Involved in Fungal Virulence to G. Mellonella

**Figure 5:**
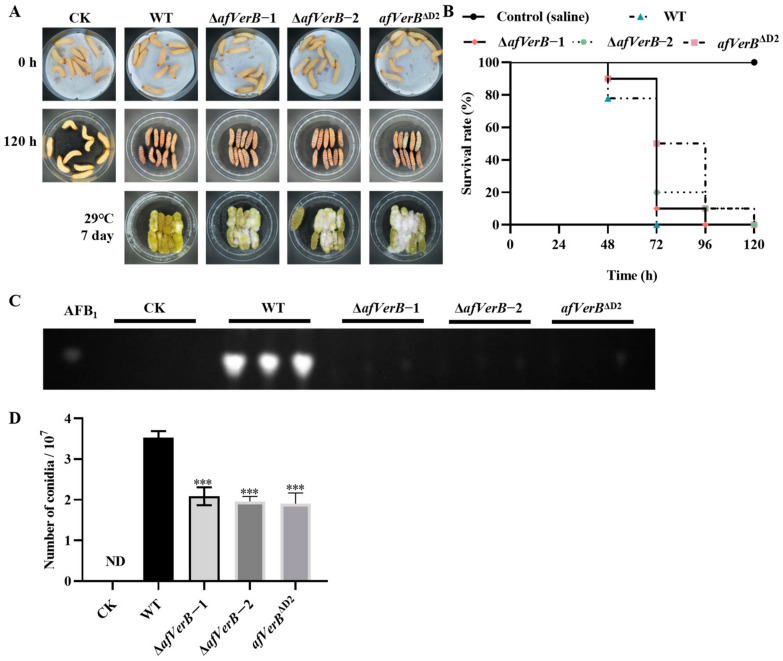
The role of AfVerB in the virulence of *A. flavus* to *G. mellonella.* (**A**) Wax moth larvae were injected with the spores of WT, ∆*afVerB*−1, ∆a*fVerB*−2, and *afVerB*^∆D2^. Spore suspensions were diluted to a concentration of 10^7^/mL with saline. Each wax moth larvae was injected with 5 μL of the diluted spore suspension, and the mortality rate of the wax moth larvae was observed over 120 hours. The dead larvae were then transferred to new *Petri* dishes and cultured in the dark for 7 d. (**B**) Survival curves of *G. mellonella* larvae injected with the above fungal strains, with larvae injected with saline serving as the negative control. (**C**) TLC analysis of the AFB_1_ produced in the dead larvae from the aforementioned groups. (**D**) Statistical analysis of the spore number on the dead larvae from the aforementioned groups. *** indicates *p* < 0.001.

To investigate the impact of *AfVerB* on the pathogenicity of *A. flavus* to animals, wax moth (*G. mellonella*) larvae were selected as the animal host. A 5 μL spore suspension (10^7^/mL) of WT, ∆*afVerB*−1, ∆*afVerB*−2, and *afVerB*^∆D2^ strains were injected into the larvae, respectively. After 120 hours of incubation and observation, it was found that all larvae in the WT group died at 72 h, while the survival rate of larvae colonized with spore suspensions of ∆*afVerB-1 and* ∆*afVerB-2* strains was at least 10% higher than that of the WT group at 72 h, and the *afVerB*^∆D2^ group even reached 50% higher than the WT group (Figure 5A,B). These results reflect that *AfVerB*, especially its second CYP domain, deeply participates in fungal virulence against wax moth larvae. The dead larvae were collected and cultured at 29 °C for 7 d, and then AFB_1_ was extracted from the dead larvae, and TLC analysis revealed that no AFB_1_ was detected in the ∆*afVerB*−1, ∆*afVerB*−2, and *afVerB*^∆D2^ groups, while large amounts of AFB_1_ were detected in the WT group (Figure 5C). The spore numbers were counted, and the results showed that the spore numbers in the ∆*afVerB*−1, ∆*afVerB*−2, and *afVerB*^∆D2^ groups were significantly reduced compared to the WT group (Figure 5D). The above results indicate that *AfVerB* plays a crucial role in fungal virulence in insects and significantly enhances the capacity of aflatoxin biosynthesis in *A. flavus*.

### 3.7. AfVerB Plays a Critical Role in the Response of A. flavus to Stresses

**Figure 6:**
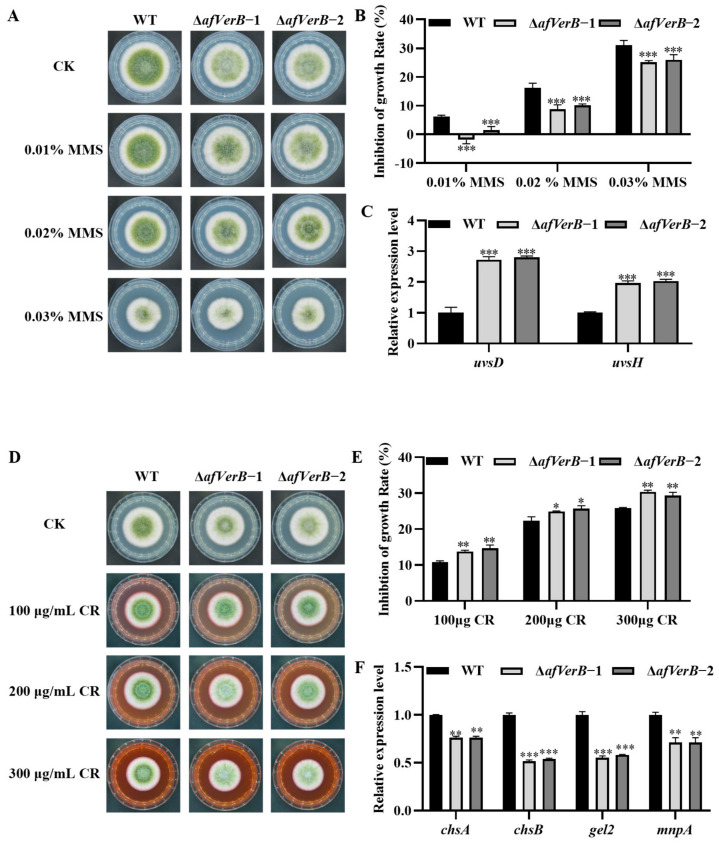
The role of AfVerB in the fungal response to DNA damage and cell wall stresses. (**A**) Growth of WT, ∆*afVerB*−1, and ∆*afVerB*−2 strains on PDA containing a series of concentrations of MMS for 4 d. (**B**) Statistical analysis of the growth inhibition rate of all the above fungal strains under MMS-mediated DNA damage stress based on panel (**A**). (**C**) The relative expression levels of *uvsd* and *uvsh* in WT, ∆*afVerB*-−1, and ∆*afVerB*−2 strains. (**D**) Growth of WT, ∆*afVerB*−1, and ∆*afVerB*−2 strains on PDA containing a series of concentrations of CR for 4 d. (**E**) Statistical analysis of the growth inhibition rates of all the aforementioned fungal strains under CR-mediated cell wall stress based on panel (**D**). (**F**) The relative expression levels of *chsA*, *chsB*, g*el2*, and *mnpA* in WT, ∆*afVerB*−1, and ∆*afVerB*−2 strains. *, **, *** means significant differences at *p* < 0.05, *p* < 0.01, and *p* < 0.001, respectively.

In order to explore the bio-functions of AfVerB in the response of *A. flavus* to various environmental stresses, the inhibitory effects of inhibitors, including KCl (mediated osmotic stress), CR (induced cell wall stress), MSB (mediated oxidative stress), and MMS (mediated DNA damage stress), was evaluated by adding them to PDA. The spore suspension (10^7^/mL) of WT, ∆*afVerB*−1, and ∆*afVerB*−2 strains was inoculated onto the medium and incubated for 4 d. The results showed that MMS (0.01%, 0.02%, and 0.03%) significantly promoted the growth of ∆*afVerB* strains compared to the WT strain (Figure 6A,B). Further RT-qPCR suggested that AfVerB inhibits fungal growth under MMS-mediated DNA damage stress by decreasing the expression of *uvsd* and *uvsh* (Figure 6C).

Under CR-mediated cell wall stress (100, 200, and 300 μg/mL), ∆*afVerB*−1 and ∆*afVerB*−2 strains were significantly inhibited compared to the WT strain (Figure 6D,E). RT-qPCR analysis implied that AfVerB may improve fungal growth under CR-mediated cell wall stress by enhancing the expression of *chsA*, *chsB*, g*el2*, and *mnpA* (Figure 6F).

To assess the role of AfVerB in the response of *A. flavus* to osmotic stress, WT, ∆*afVerB*−1, and ∆*afVerB*−2 strains were inoculated onto PDA containing various concentrations of KCl (0.5, 1.0, and 1.5 M) for 4 d. The results showed that the growth inhibition rates of ∆*afVerB*-1 and ∆*afVerB*-2 strains were significantly increased compared to that of the WT strain. RT-qPCR suggested that AfVerB may enhance fungal growth under KCl-mediated osmotic stress by improving the expression of *skn7*, *sskA*, and *sskB* (Figure S3A–C). Oxidative stress mediated by MSB was also evaluated, and the results showed that AfVerB may decrease fungal growth under MSB-mediated oxidative stress by suppressing the expression of *catA* and *catB* (Figure S3D–F). The above findings indicate that AfVerB plays a key role in the response of *A*. *flavus* to various environmental stresses.

### 3.8. AfVerB Is Involved in the Response of A. flavus to Antifungal Drugs

**Figure 7:**
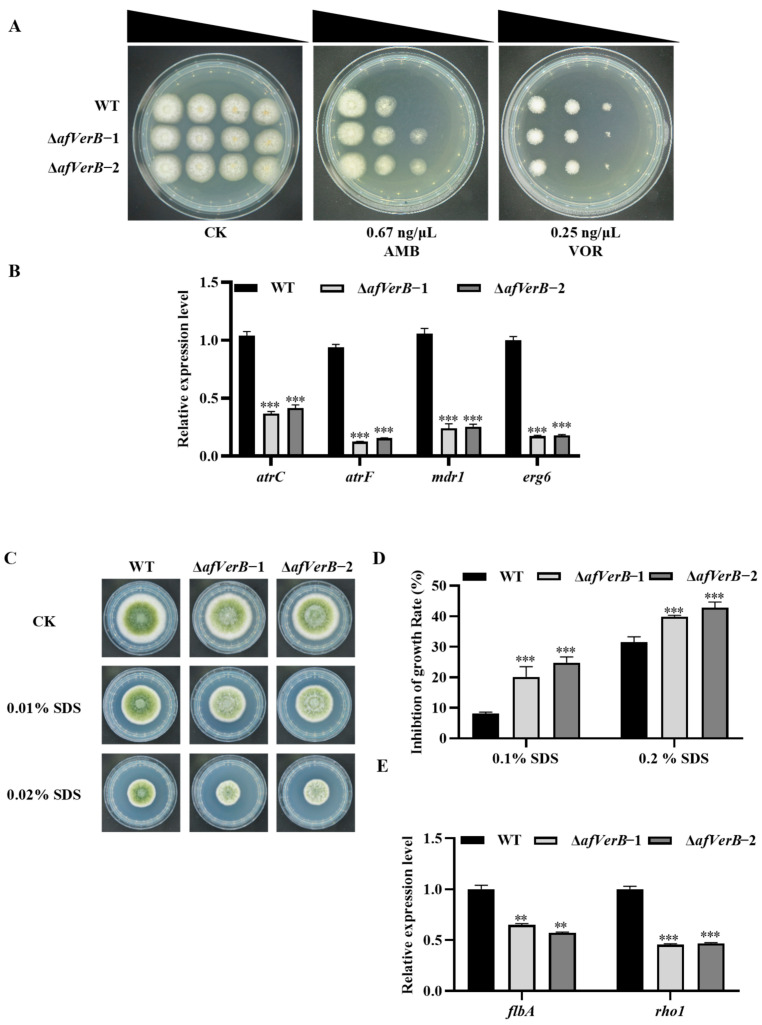
AfVerB plays an important role in drug sensitivity regulation by maintaining plasma membrane stability. (**A**) Spores of WT, ∆*afVerB*−1, and ∆*afVerB*−2 were diluted to 2 × 10^6^, 2 × 10^5^, 2 × 10^4^, and 2 × 10^3^ and inoculated onto MHA medium containing 0.67 μg/mL AMB and 0.25 μg/mL VOR. The growth state was observed after 48 h of incubation at 37 °C. (**B**) Statistical analysis of the relative expression levels of *mdr1*, *atrC*, *atrF*, and *erg6* in WT, ∆*afVerB*−1, and ∆*afVerB*−2 strains. (**C**) Growth of WT, ∆*afVerB*−1, and ∆*afVerB*−2 strains on PDA containing a series of concentrations of SDS for 4 d. (**D**) Statistical analysis of the growth inhibition rate of all the aforementioned fungal strains under SDS mediated cellular membrane stress based on panel (**C**). (**E**) Statistical analysis of the relative expression levels of *rho1* and *flBA* in WT, ∆*afVerB*−1, and ∆*afVerB*−2 strains. ** and *** means significant differences at *p* < 0.01, and *p* < 0.001, respectively.

In order to investigate the bio-function of *afVerB* in fungal drug resistance, the spore suspension of WT, ∆*afVerB*−1, and ∆*afVerB*−2 strains was diluted to 2 × 10^6^, 2 × 10^5^, 2 × 10^4^, and 2 × 10^3^ conidia/mL, and inoculated on MHA medium containing final concentrations of 0.67 ng/μL AMB and 0.25 ng/μL VOR, respectively. It was found that the ∆*afVerB*−1 and ∆*afVerB*−2 strains were more resistant to AMB but more sensitive to VOR compared to the WT strain (Figure 7A). Further RT-qPCR analysis was conducted on the drug efflux genes (*atrC*, *atrF*, and *mdr1*) and ergosterol biosynthesis-related genes (*erg6*). The results showed that *atrC*, *atrD mdr1*, and *erg6* expression levels were significantly down-regulated in the absence of AfVerB. These results suggest that AfVerB enhances VOR resistance by increasing drug efflux through upregulating the expression of *mdr1*, *atrC*, and *atrF*, while promoting fungal susceptibility to AMB by increasing ergosterol synthesis via upregulating the expression of *erg6* (Figure 7B).

Ergosterol is a key component of the fungal cell membrane [30]. Antifungal agents bind to ergosterol in the fungal cell membrane, thereby increasing the permeability of the cell membrane, which inhibits fungal growth and proliferation [31,32]. In light of these findings, we speculated that AfVerB plays an important role in keeping the dynamic equilibrium of fungal plasma membrane. Therefore, further research on the bio-function of AfVerB in cell membrane homeostasis is warranted. WT, ∆*afVerB*−1, and ∆*afVerB*−2 strains were inoculated on PDA under SDS-mediated cell membrane stress (0.01% and 0.02%), and the results showed that the growth inhibition rates of ∆*afVerB*−1 and ∆*afVerB*−2 strains were significantly increased compared to that of the WT strain (Figure 7C,D). RT-qPCR analysis implied that AfVerB may be involved in the maintenance of cell membrane stability by enhancing the expression of *flba* and *rho1* (Figure 7E). The above results suggest that AfVerB is a key regulator in maintaining plasma membrane stability and may be one of the important potential candidates in regulating fungal drug sensitivity in the fight against drug-resistant invasive Aspergillosis.

## 4. Discussions

AfVerB is a pivotal gene in the biosynthetic pathway of aflatoxin, encoding an enzyme that participates in the synthesis of aflatoxin [33]. AfVerB is involved in the conversion from Versicolorin A to Demethylsterigmatocystin, which is crucial for the subsequent synthesis of aflatoxin [15,16,17,34]. Affinin and the ethanol extract from *Heliopsis longipes* can significantly inhibit the biosynthesis of aflatoxin in *A. parasiticus* through downregulating multiple genes in the aflatoxin gene cluster, including *afVerB* [35]. Given the severe impact of aflatoxin on crops and human health, it is imperative to investigate the role of the toxin-related gene *afVerB* in the growth, development, AFB_1_ synthesis, sclerotial formation, and pathogenicity of *A. flavus*. However, the regulatory role of *afVerB* in the phenotype, secondary metabolism, and pathogenicity of *A. flavus* has not been deeply reported. Therefore, this study was designed to unveil the bio-function of AfVerB in *A. flavus*.

In the analysis of the growth and development of *A. flavus*, we found that the deletion of *AfVerB* significantly affected fungal growth rate and conidiation, and both conserved CYP domains were significantly involved in the process (Figure 2A,C). A similar phenotype was observed in the colonization of peanuts and corn (Figure 4A,B,D,E), as well as in the colonization of wax moths (Figure 5A). In *Aspergillus* species and related fungi, the formation of conidiospores is primarily regulated by the central developmental pathway composed of *abaA*, *brlA*, and *wetA* [36]. *brlA* begins to be expressed in the early stage of conidiophore development, controlling the formation of conidiophores [37]. *abaA* functions in the middle stage of conidial development by activating the expression of the downstream gene *wetA*, thereby ensuring normal conidial development [38]. *wetA*, in the late stage of conidial development, regulates the synthesis of the conidial cell wall, conidial and hyphal development, and the formation of pigments, ensuring the normal maturation of conidia [39]. In this study, RT-qPCR revealed that AfVerB upregulates the expression levels of the transcription factors *abaA* and *brlA*, indicating that, through its CYP domains, AfVerB is involved in the regulation of the early and middle stage of conidia formation (including conidiophore and phialide formation), but does not participate in *wetA* mediated late stage of conidia development (Figure 2D).

Sclerotia is a structure of *Aspergillus* species’ adaptation to adverse environmental conditions, and it is also the structure responsible for the sexual reproduction of *A. flavus* [40,41]. In this study, the deletion of *afVerB* significantly impacted the formation of sclerotia in *A. flavus*. Similarly, the deletion of either conserved domain markedly reduced the number of sclerotia (Figure 2E,F). *nsdC* and *nsdD* are key regulatory factors in sclerotia formation [42]. The deletion of AfVerB or its two CYP domains resulted in the downregulation of transcriptional factors *nsdC* and *nsdD* (Figure 2G). The above results reflect that through positively regulating both *nsdC* and *nsdD*, AfVerB, mainly through its CYP domains, enhances sclerotia formation and may promote genetic variation in *A. flavus* by facilitating sexual reproduction, thereby enhancing the environmental adaptability of this pathogenic fungus.

This study revealed that the ability to synthesize aflatoxins was lost in different carbon source media after the deletion of *afVerB* or its second CYP domain (Figure 3A). Further RT-qPCR analysis revealed that AfVerB dramatically inhibits the production of AFB_1_ by downregulating the expression of the aflatoxin gene cluster, including *aflR*, *aflG*, and *aflP* (Figure 3B). AflR is the initial transcriptional factor that activates aflatoxin synthesis, and as the important regulator in the aflatoxin gene cluster, the activity of *aflP* and *aflG* are regulated by *aflR* [43,44]. In the aflatoxin gene cluster, *afVerB* and *aflG* are closely related, and the ability of *aflG* to participate in aflatoxin biosynthesis is lost when the *afVerB* gene is deleted [45]. Additionally, studies have shown that the CYP domain plays an important role in the biosynthesis of aflatoxins [46,47]. This study found that the deletion of the first CYP domain (*afVerB*^D1^) does not affect the synthesis of aflatoxins, while the deletion of its second CYP domain (*afVerB*^D2^) results in the total loss of toxin synthesis ability (Figure 3A). It inferred that the second CYP domain of AfVerB plays a key role in aflatoxin biosynthesis regulation. The same phenotype was observed in the colonization of peanuts and corn, as well as in the colonization of immune-competent *G. mellonella* [48]. These results suggest that AfVerB regulates the synthesis of afltoxins in *A*.*flavus* through its second CYP domain.

External conditions have a certain impact on the growth and development of *Aspergillus* [49]. The capacity of pathogenic fungi to cope with various environmental stresses is closely related to their ability to infect hosts. Therefore, it is important to explore the role of AfVerB in fungal sensitivity against various stresses. We found that AfVerB is involved in the osmotic stress mediated by KCl (Figure S3), the oxidative stress mediated by MSB (Figure S3), the DNA damage stress mediated by MMS (Figure 6), and the cell wall stress mediated by CR (Figure 6). The absence of *skn7* leads to increased sensitivity of cells to high osmotic pressure, and *skn7* regulates the Hog1 MAPK pathway to respond to high osmotic pressure in *S*. *cerevisiae* [50]. SskA and SskB are high osmotic regulators involved in the response to osmotic stress and the regulation of cell growth [50,51,52]. We have discovered that AfVerB exerts a significant influence on osmotic stress response by upregulating the expression levels of *skn7*, *sskA*, and *sskB* (Figure S3C), thereby playing a crucial role in maintaining osmotic homeostasis. In the context of DNA damage stress mediated by MMS, the genes *uvsd* and *uvsh* are involved in the nucleotide excision repair (NER) pathway, which is essential for DNA repair, genomic stability, and cellular survival [53,54]. Our findings indicated that AfVerB downregulates the expression of *uvsd* and *uvsh*, and the growth inhibition rate of MMS on the *afVerB*-deficient strains (∆*afVerB*−1, and ∆*afVerB*−2) is significantly lower than that of the wild-type (WT) strain (Figure 6A–C). This suggests that AfVerB is involved in the DNA damage stress response by downregulating the activity of *uvsd* and *uvsh*. Genes *catA* and *catB* are two important catalase genes that play key roles in the antioxidant defense and secondary metabolism of *A. flavus* [55]. We observed that AfVerB downregulates the expression of *catA* and *catB* under the stress of MSB, and the growth inhibition rate of the *afVerB*-deficient strain (∆*afVerB*-1, and ∆*afVerB*-2) is significantly lower than that of the WT strain (Figure S3D–F). This indicated that AfVerB is involved in the MSB-mediated oxidative stress response by downregulating the activity of *catA* and *catB*. Genes *chsA* and *chsB* are chitin synthase genes that play important roles in cell wall integrity [56,57]. The g*el2* gene encodes β (1-3) glucan synthase, which actively participates in fungal cell wall biosynthesis, while *mnpA* is crucial for cell wall integrity and developmental patterning [58,59]. We found that AfVerB upregulates the expression levels of *chsA*, *chsB*, g*el2,* and *mnpA* under CR-mediated cell wall stress, and the growth inhibition rate of the *afVerB*-deficient (∆*afVerB*−1, and ∆*afVerB*−2) strain is significantly higher than that of the WT strain (Figure 6D–F). These results demonstrate that AfVerB plays a vital role in maintaining cell wall stability by up-regulating the expression levels of *chsA*, *chsB*, g*el2,* and *mnpA*.

VOR primarily inhibits the 14α-demethylation of fungal cytochrome P450, blocking ergosterol synthesis and disrupting fungal cell membrane stability, leading to the leakage of cellular contents and thereby inhibiting fungal growth and reproduction [31]. AMB destroys the integrity of the fungal cell membrane through multiple mechanisms, increasing intracellular oxidative damage, and effectively inhibiting fungal growth and reproduction [60]. The role of AfVerB in fungal sensitivity to antifungal drugs was explored in this study. Compared with the WT, we found that the mutants lacking AfVerB were more sensitive to VOR, but more resistant to AMB. Mitochondrial-deficient strains of *A. fumigatus* have developed resistance to itraconazole, mainly due to the high expression of the drug efflux pump gene *mdr1* [61]. ATP-binding cassette (ABC) transporters are major contributors to antifungal drug resistance in pathogenic fungi [62]. In *A. nidulans*, two novel ABC transporter-encoding genes *atrC* and *atrD* were identified, and evidence was found that *atrD* is involved in multidrug transport and antibiotic production [63]. In *A. fumigatus*, the C2H2 transcription factor ZfpA can significantly upregulate the ABC transporter *atrF*, thereby enhancing its resistance to azole drugs [64]. *Erg6* plays a crucial role in the ergosterol biosynthesis pathway. In relevant studies, it has been found that in AMB-resistant strains, the mRNA levels of *erg6* are decreased, making it a potential target for reducing AMB sensitivity [65,66]. In this study, RT-qPCR analysis revealed that AfVerB confers fungal resistance to VOR by upregulating the expression of *atrC*, *atrF*, and *mdr1*, while enhancing fungal sensitivity to AMB by upregulating the expression of the *erg6* gene (Figure 7A,B). Therefore, our results suggest that AfVerB is involved in the regulation of fungal resistance to VOR and fungal sensitivity to AMB by modulating the integrity of the fungal cell membrane.

The key role of AfVerB in the sensitivity of *A. flavus* to VOR and AMB reflected that AfVerB is deeply involved in the stability of the plasma membrane. To verify the above viewpoint, the role of AfVerB in the fungal response against SDS-mediated plasma membrane stress was explored. The results showed that AfVerB is a very important element in maintaining the homeostasis of the plasma membrane (Figure 7C,D). SDS-mediated stress triggers a kinase cascade response known as the cell wall integrity (CWI) pathway, which responds to disturbances in the cell wall and membrane to maintain cellular integrity [67]. G proteins, as key regulatory proteins in the CWI pathway, act in concert with *flbA* to modulate fungal morphogenesis [68]. The deletion of the r*ho1* gene leads to the rupture of the plasma membrane, cytoplasmic leakage, and ultimately cell necrosis [69]. In this study, further signaling pathway analysis through RT-qPCR revealed that AfVerB maintains plasma membrane stability by upregulating the expression levels of *flbA* and *rho1* under SDS-mediated cell membrane stress (Figure 7E). Additionally, RT-qPCR analysis revealed that *afVerB* is downregulated by the chromatin remodeling factor (CRF) AflArp5 and AflArp8, and upregulated by the CRF Aflarp9 and AflRsc1 (Figure S4). Based on the above findings, we speculated that AfVerB is deeply involved in maintaining the stability of the cell membrane, playing a key role in the fungal response to antifungal drugs. In view of the above deduction, AfVerB may serve as a potential target in the fungal drug-resistance field.

In summary, we have explored and elucidated the biological functions of AfVerB, including its role in fungal morphogenesis, mycotoxin biosynthesis, regulation of fungal virulence, and response to a series of environmental stresses, as well as its involvement in fungal drug resistance. We also revealed that its CYP domains, especially the second CYP domain, play a crucial role in the process of AfVerB implementing its bio-functions. These findings shed light on the regulatory mechanisms of AfVerB in fungal virulence and secondary metabolism. However, how the methyltransferase AflIme4 regulates the action of AfVerB remains unknown. Further investigation into the role of AflIme4-mediated mRNA methylation in the biological function of AfVerB would reveal the complicated regulatory mechanism of fungal virulence and secondary metabolism at the epigenetic level. This study provides new insights into the development of potential strategies against pathogenic filamentous fungi in the future.

## Data Availability

The authors confirm that the data supporting the findings of this study are available within the article and its Supplementary Materials.

## Supplementary Materials

The following supporting information can be downloaded at: https://www.mdpi.com/article/10.3390/jof11040293/s1. Table S1: Strains used in this study; Table S2: The media used in this study; Table S3:Primers used in this study; Figure S1: The scheme of fungal strain construction; Figure S2: The construction of AfVerB mutants and the sequencing results of CYP domain deletion strains; Figure S3:The role of AfVerB in fungal response to osmotic and oxidative stresses. Figure S4:The impact of Afverb on chromatin remodeling factors.
