# Supplementary material for: Molecular Mechanism of Aflatoxin B1 Synthesis Related AfVerB Regulating the Development, AFB1 Biosyntheis and Virulence of Aspergillus flavus Mainly Through Its CYP Domain
Source: J Fungi (Basel). 2025 Apr 9;11(4):293. doi: 10.3390/jof11040293 (PMC12028525; doi:10.3390/jof11040293)
Supplement: Supplementary file 1 [file jof-11-00293-s001.zip › jof-3472994-supplementary.pdf]

Table S1. Strains used in this study.

Strain	Characterization	Source
A. *flavus*·CA14 PTS	Δ*ku70*,Δ*pyrG*	Purchased from FGSC
Wild type (WT)	Δ*ku70*,ΔpyrG:: *pyrG*	Prepared in our lab
Δ*afVerB*-1	Δ*ku70*,Δ*afVerB*-1:: *pyrG*	This study
Δ*afVerB*-2	Δ*ku70*,Δ*afVerB*-2:: *pyrG*	This study
afVerB$^{\Delta D1}$	Δ*ku70*,Δ*afVerB*:: *pyrG*, *D1*:: *pyrG*	This study
afVerB$^{\Delta D2}$	Δ*ku70*,Δ*afVerB*:: *pyrG*, *D2*:: *pyrG*	This study

Table S2. The media used in this study

Types of media	Components of media
PDA	Potato Dextrose Agar (BD USA) 39 g/L
PDB	Potato Dextrose Broth (BD USA) 24 g/L
MHA	Müller-Hinton Agar (OXOID) 38 g/L
YES	150 g/L sucrose, 20 g/L yeast powder, 1 g/L $MgSO_4$, 1.5% agar
GMM	6 g/L $NaNO_3$, 0.52 g/L $MgSO_4$, 1.52 g/L KH_2PO_4, 10 g/L glucose, 0.52 g/L KCl, 1 mL/L trace elements, 1.5% agar
CM	6 g/L TPYPONE, 6 g/L yeast extract, 10 g/L sucrose, 1.5% agar
Resuscitation culture medium	342.4 g/L sucrose, 1 g/L K_2HPO_4, 0.5 g/L KCl, 0.5 g/L $MgSO_4$, 0.01 g/L $FeSO_4$, 1.84 g/L ammonium tartrate, 1.5% agar to the lower layer medium, 0.5% agar to the upper layer medium.

Table S3. Primers used in this study.

Primer name	Sequence (5' to 3')	Fragment amplified
Δ*afVerB*-AF	TCCCTCCTCGGTGTATATC	Construction of Δ*afVerB*
Δ*afVerB*-AR	GGGTGAAGAGCATTGTTTGAGGCCTAGTTCGAGGCGTGAAG	
Δ*afVerB*-BF	GCATCAGTGCCTCCTCTCAGACGGCATACAGTCATAGAGTCTCA	
Δ*afVerB*-BR	GCCGTTGGTACTATGGTATAA	
Δ*afVerB*-NF	GGCTCCAGTATTCTACGTCA	
Δ*afVerB*-NR	GGCAGACGGTCAACGATAT	
afVerB$^{\Delta D1}$-P1	ACAAGAAACCCACAAGTCCACG	Construction of *afVerB*$^{\Delta D1}$
afVerB$^{\Delta D1}$-P2	CAGATCTTCTGATCGAGGAAGTTGCCTGGACCAGGATATCCACGGAGT	
afVerB$^{\Delta D1}$-P3	ACTCCGTGGATATCCTGGTCCAGGCAACTTCCTCGATCAGAAGATCTG	
afVerB$^{\Delta D1}$-P4	GGTGAAGAGCATTGTTTGAGGCTCTACTACTCATTGCGAGGTTCCAGC	
afVerB$^{\Delta D1}$-BF	GCATCAGTGCCTCCTCTCAGACGGCATACAGTCATAGAGT	

	CTCA	
afVerB$^{\Delta D1}$-BR	GCCGTTGGTACTATGGTATAA	
afVerB$^{\Delta D2}$-P1	ACAAGAAACCCACAAGTCCA	
afVerB$^{\Delta D2}$-P2	GCCCTGCTCTGCCAGCCAATATGATCGTAAAGGATGGATCCA	
afVerB$^{\Delta D2}$-P3	CATCCTTTACGATCATATTGGCAGAGCAGGGCATTT	Construction of *afVerB*$^{\Delta D2}$
afVerB$^{\Delta D2}$-P4	GCCCTGCTCTGCCAGCCAATATGATCGTAAAGGATGGATCCA	
afVerB$^{\Delta D2}$-BF	GCATCAGTGCCTCCTCTCAGACGGCATACAGTCATAGAGTCTCA	
afVerB$^{\Delta D2}$-BR	GCCGTTGGTACTATGGTATAA	
PyrG-F	GCCTCAAACAATGCTCTTCACCC	To amplify pyrG
PyrG-R	GTCTGAGAGGAGGCACTGATGC	
Δ*afVerB*-OF	GCGATGTATCTGCTGTGTCT	To verify the existence of ORF
Δ*afVerB*-OR	ACCTGGTCGGTCGTGAA	
Δ*afVerB*-NF	GGCTCCAGTATTCTACGTCA	To verify the existence of A
P801-R	CAGGAGTTCTCGGGTTGTCG	
P1020-F	ATCGGCAATACCGTCCAGAAGC	To verify the existence of B
Δ*afVerB*-NR	GGCAGACGGTCAACGATAT	
P1020-F	ATCGGCAATACCGTCCAGAAGC	To verify the existence of AP and BP
P801-R	CAGGAGTTCTCGGGTTGTCG	
afVerB$^{\Delta D1}$-D1F	AGCGTGGGGATGGAATG	To verify the D1 of ORF
afVerB$^{\Delta D1}$-D1R	GGCGTGAGTAAAGTTGTAGTCG	
afVerB$^{\Delta D1}$-D1AF	GGCTCCAGTATTCTACGTCA	To verify the D1 of A
P801-R	CAGGAGTTCTCGGGTTGTCG	
afVerB$^{\Delta D1}$-D1BF	TGCTCGGCTACTTCAACTCAT	To verify the D1 of B
afVerB$^{\Delta D1}$-D1BR	CCACGCCCTTACCTTCTTC	
afVerB$^{\Delta D2}$-D2F	TTTTCATTCTCCGACAAAGG	To verify the D2 of ORF
afVerB$^{\Delta D2}$-D2R	TAACAGCCAAGTACACCCACT	
afVerB$^{\Delta D2}$-D2AF	GGCTCCAGTATTCTACGTCA	To verify the D2 of A
P801-R	CAGGAGTTCTCGGGTTGTCG	

afVerB$^{\Delta D2}$-D2BF	TGCTCGGCTACTTCAACTCAT	To verify the D2 of B
afVerB$^{\Delta D2}$-D2BR	CCACGCCCTTACCTTCTTC	
abaA-qF	TCTTCGGTTGATGGATGATTTC	To detect the transcription level of conidia formation genes
abaA-qR	CCGTTGGGAGGCTGGGT	
brlA-qF	GCCTCCAGCGTCAACCTTC	
brlA-qR	TCTCTTCAAATGCTCTTGCCTC	
wetA-qF	ATTCATCATTTTCCTCAACGACC	
wetA-qR	TTGACGGGTTTGGGGACAT	
nsdD-qF	GGACTTGCGGGTCGTGCTA	To detect the transcription level of sclerotia formation genes
nsdD-qR	AGAACGCTGGGTCTGGTGC	
nsdC-qF	GCCAGACTTGCCAATCAC	
nsdC-qR	GGACTTGCGGGTCGTGCTA	
aflR-qF	AAAGCACCCTGTCTTCCCTAAC	To detect the transcription level of aflatoxin biosynthesis genes
aflR-qR	GAAGAGGTGGGTCAGTGTTTGTAG	
aflG-qF	GAAGAGTTCCTGCCTGAG	
aflG-qR	CATCTGCGTAGTATCTAATCG	
aflP-qF	CGATGTCTATCTTCTCCGATCTATTC	
aflP-qR	TCTCAGTCTCCAGTCTATTATCTACC	
uvsD-qF	CATCCGATCAAATCAGGCG	To detect the transcription level of genes related to DNA damage stress mediated by MMS
uvsD-qR	CCTCTTCCGTTTACCACCCA	
uvsH-qF	AACCCCGTTATTACCTCGTG	
uvsH-qR	CTTCAGTTTGCCGCTCCA	
chsA-qF	TTTCCACATTCTCACGACAT	To detect the transcription level of genes related to cell wall
chsA-qR	TACCAATGTTGCGAGGTAAA	
chsB-qF	GTATCTCTCGGGAATGATCG	
chsB-qR	GAAGAAGTAGAGACCGACAC	
gel2-qF	ATGTAGTCCTTCATGTCACG	
gel2-qR	CTTCAAGAACTACCCGAACA	
mnpA-qF	GACATCATCTCTGGCATCAG	

mnpA-qR	TGATATCATCACCGCTCTTG	stress mediated by CR
skn7-qF	TCTCTGATCGTTATGCACAG	To detect the transcription level of genes related to osmotic stress mediated by KCl
skn7-qR	GTTGACGAGCATCTACTGAT	
sskA-qF	ACCAAAGCTAACACATCCAT	
sskA-qR	CTGTGTCTGGGAAAGATGAA	
sskB-qF	CGGAGATTCTCGAACAAGAT	
sskB-qR	CACATGCACGTTACAAATCA	
catA-qF	CGACGAACCTAGAACCTACC	To detect the transcription level of genes related to Oxidative Stress mediated by MSB
catA-qR	AAACACTTGGTCTTCTTGCC	
catB-qF	CGAACGTGTTCCTGAACG	
catB-qR	GCCAACAATATCGAAGTTACC	
catC-qF	GAAAGAGTTGTCCATGCCA	
catC-qR	CAGAAAACGGGTGTGTGAT	
flbA-qF	TCCACGTTCACCAGCACTAC	To detect the transcription level of genes related to cell membrane stress mediated by SDS
flbA-qR	TACCTCCAATGGCCGAGAGA	
rho1-qF	TACCCCGACTCTCACGTCAT	
rho1-qR	TCTTGCGAACTTCCTCACCC	
atrC-qF	CGCTGCCTGAGAAGGAAAGA	To detect the transcription level of genes related to drug efflux mediated by VOR and AMB
atrC-qR	GGATATGTCGCTCCTCCCAC	
atrF-qF	CGGTCCTGGTCAAGCACAAA	
atrF-qR	TGCGGTCATAACCATTGTCG	
mdr1-qF	GCACTACCGCTATTCACAATCC	
mdr1-qR	CGCGGATCTTTTGGCTGATG	
erg6-qF	TGGGTGTGGTCAATAACTACTACG	
erg6-qR	ACCTGGCACCCAGTAAATCG	

Tubulin-F<br>*Tubulin*-R	GAAGGTGGAGGACATCTTG<br>ACTCTTTCCGTGCCGTCTC	To detect the transcription level of control gene

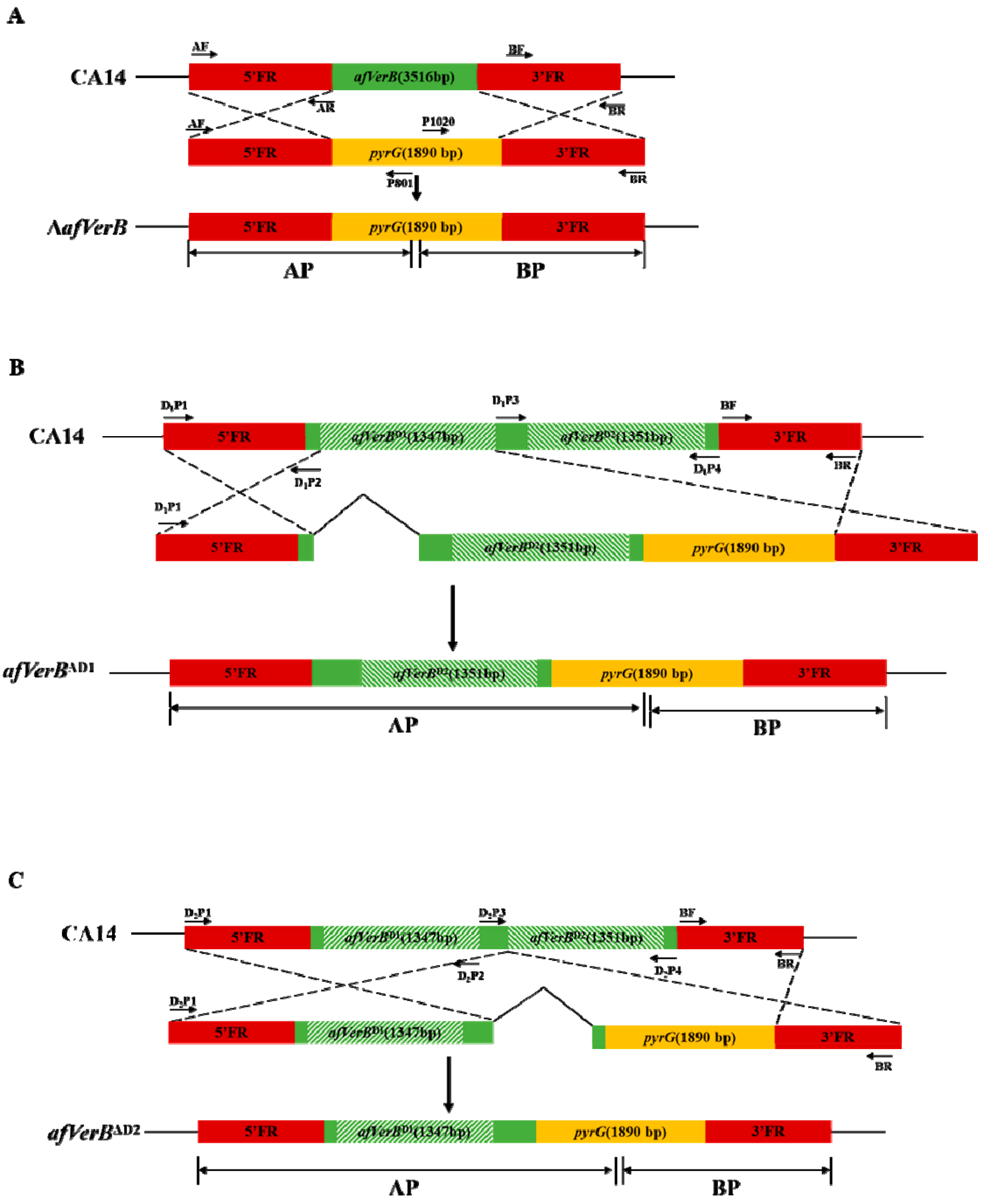

Figure S1. The scheme of fungal strain construction. (A)The construction principle of *afVerB* knock out strain (Δ*afVerB*). (B)The principle of constructing the domain 1 (D1) knock out strain $afVerB^{\Delta D1}$.(C)The principle of constructing the domain 2 (D2) knock out strain $afVerB^{\Delta D2}$.

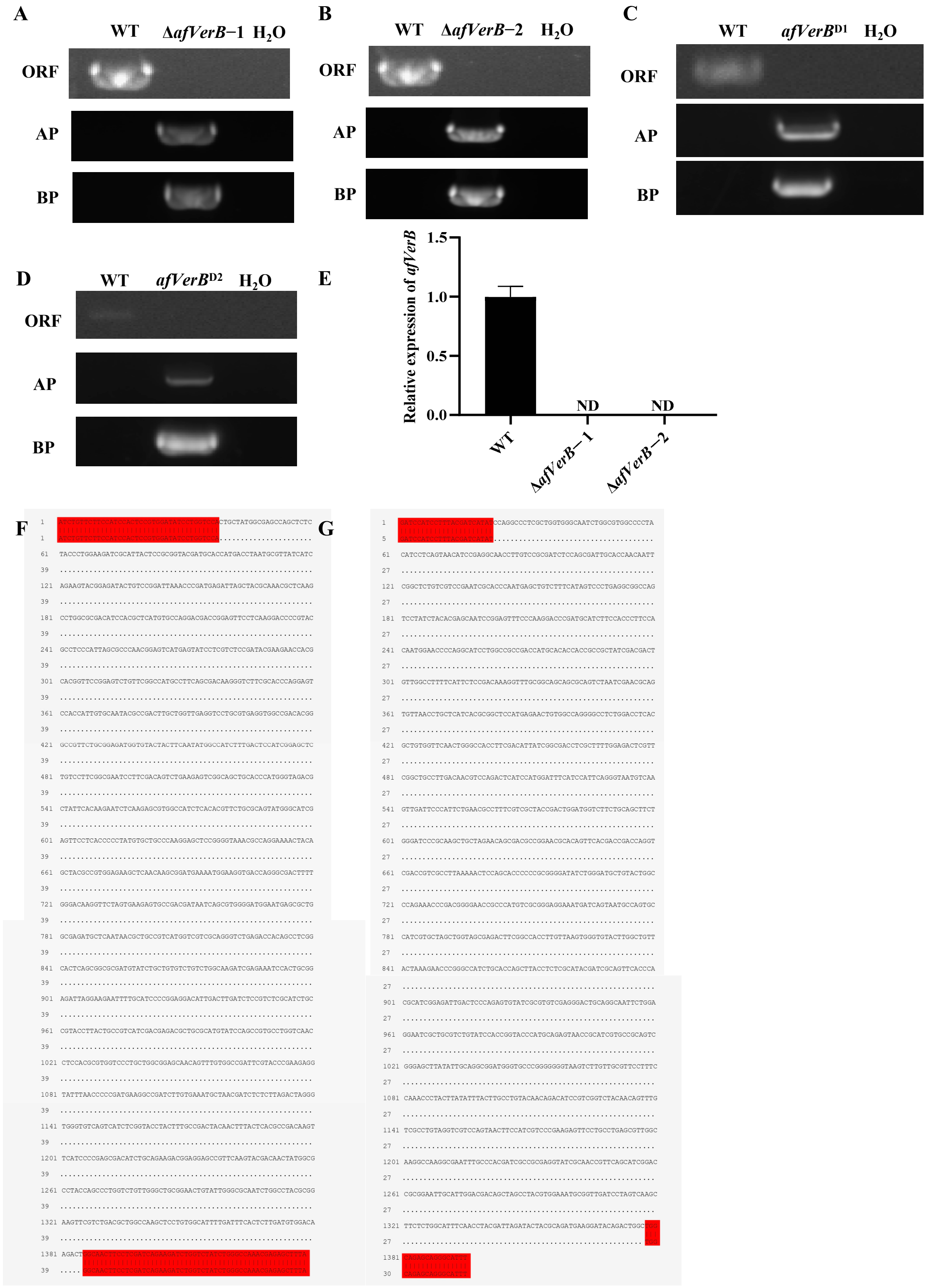

Figure S2 The construction of AfVerB mutants and the sequencing results of CYP domain deletion strains. **(A. B)** Verification of Δ*afVerB*-1 and Δ*afVerB*-2 mutants by diagnostic-PCR. Since *afVerB* was replaced by *PyrG*, fragments A and B could be amplified from the genome, but the ORF fragment could not

be amplified. (**C. D**). The domain deletion mutants *afVerB*$^{\Delta D1}$ and *afVerB*$^{\Delta D2}$ were verified by diagnostic-PCR. Due to the deletion of the D1 or D2 domain, fragments D1 or D2 could not be amplified in corresponding mutant, whereas fragments A and B could be successfully amplified. D1 is represented for the first CYP domain, D2 is represented for the second CYP domain. **(E)** The relative expression levels of *afVerB* in WT, Δ*afVerB*-1 and Δ*afVerB*-2 were tested by RT-qPCR. ND indicates not signal. (F) The sequencing result of *afVerB*$^{\Delta D1}$. (G) The sequencing results of *afVerB*$^{\Delta D2}$. Sequencing were carried out in Fuzhou Sunya Biotechnology Co., LTD (Fuzhou, China).

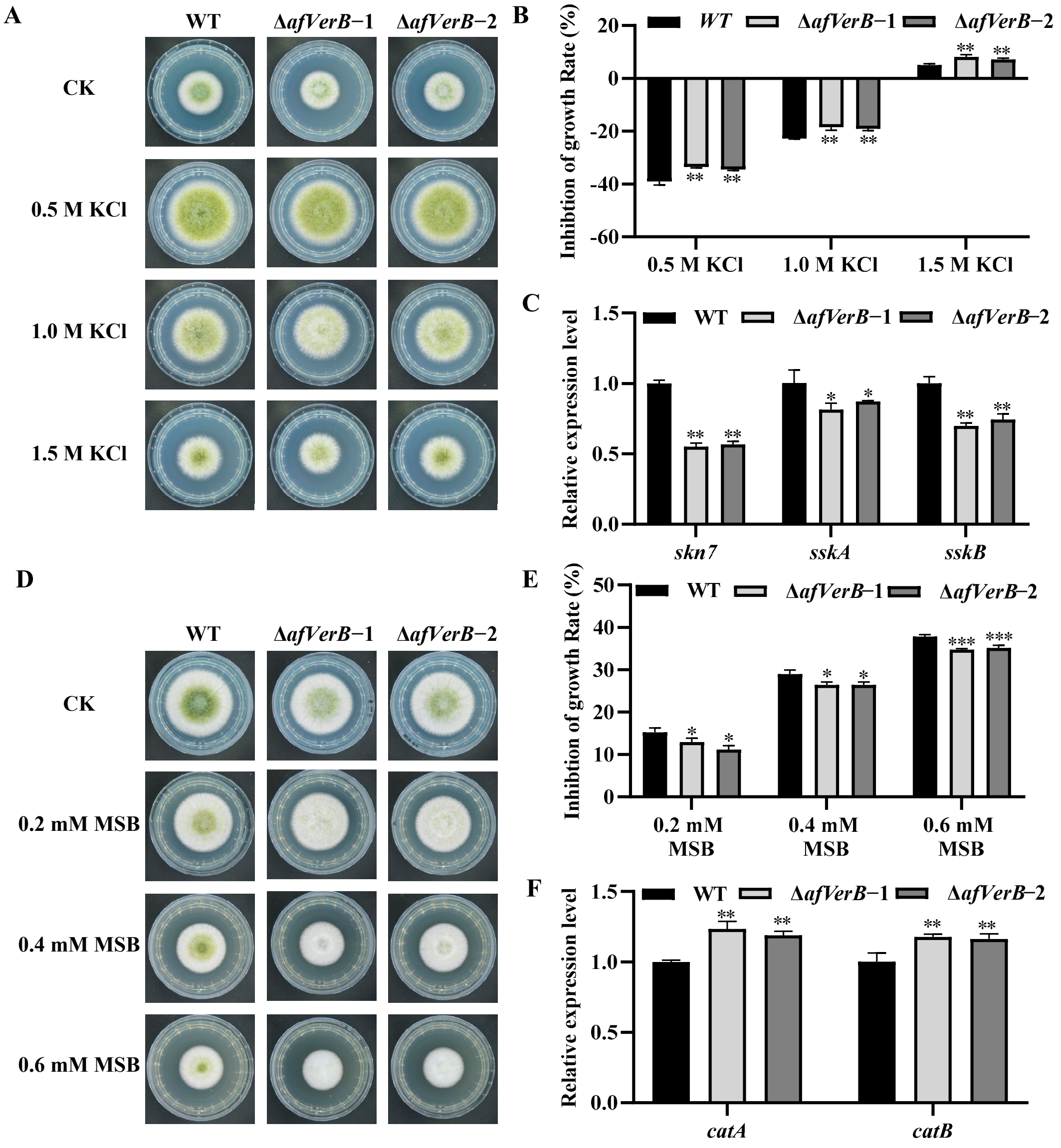

Figure S3. The role of AfVerB in fungal response to osmotic and oxidative stresses.

(A) Growth of WT, Δ*afVerB*-1, and Δ*afVerB*-2 strains on PDA containing a series of concentrations of KCl for 4 d. **(B)** Statistical analysis of the growth inhibition rate of all the above fungal strains under KCl mediated osmotic stress based on Panel A. **(C)** The relative expression levels of *skn7*, *sskA* and *sskB* in WT, Δ*afVerB*-1, and Δ*afVerB*-2 strains. **(D)** Growth of WT, Δ*afVerB*-1 and Δ*afVerB*-2 strains on PDA containing a series of concentrations of MSB for 4 d. **(E)** Statistical analysis of the growth inhibition rate of all the aforementioned fungal strains under MSB mediated oxidative stress based on the Panel D. **(F)** The relative expression levels of *catA* and *catB* in WT, Δ*afVerB*-1 and Δ*afVerB*-2 strains. *,**,*** means significant difference P<0.05, P<0.01, P<0.001.

A

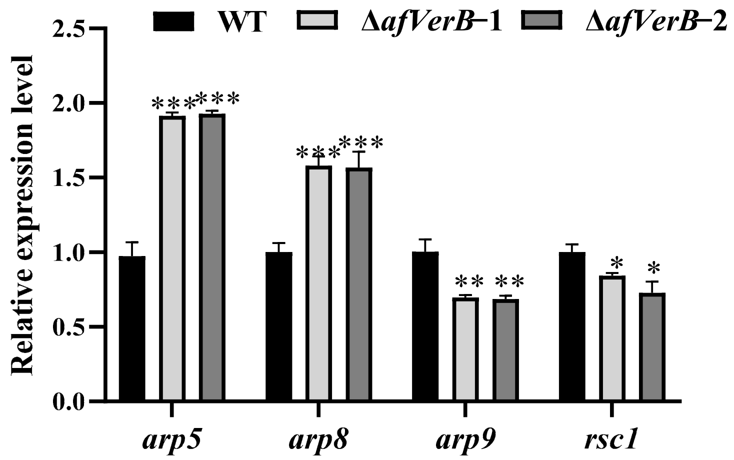

Figure S4. The impact of Afverb on chromatin remodeling factors. **(A)** The relative expression levels of a*rp5*, *arp8* ,*arp9* and *rsc1* in WT, Δ*afVerB*-1 and Δ*afVerB*-2 strains. *,**,*** means significant difference P<0.05, P<0.01, P<0.001.
